# Influence of Anomalous Changes in the Crystal Structure on the Transport Properties of YbNi_1−x_Cu_x_Al Series of Alloys

**DOI:** 10.3390/ma15051688

**Published:** 2022-02-24

**Authors:** Daniel Rojas Pupo, Flávio Guimarães Gandra, Luis Fernández Barquín

**Affiliations:** 1 Departamento de Estructuras y Física de Edificación, ETSAM, Universidad Politécnica de Madrid, 28040 Madrid, Spain; 2 Instituto de Física, Universidade Estadual de Campinas, Campinas 13083-970, Brazil; gandra@ifi.unicamp.br; 3 Departamento CITIMAC, Facultad de Ciencias, Universidad de Cantabria, 39005 Santander, Spain; barquinl@unican.es

**Keywords:** Yb alloys, intermediate valence, heavy fermion

## Abstract

Results of the transport properties of the YbNi_1−x_Cu_x_Al (x = 0, 0.2, 0.5, 0.8 and 1.0) series of alloys are reported. The previous analysis of X-ray diffraction patterns indicates that all compounds crystallize in the hexagonal ZrNiAl structure with a linear behavior of the unit cell volume as a function of the Cu concentration (x). This is not found in the unit cell parameters, showing a discontinuity between x = 0.5 and 0.8. Such discontinuities affect the behavior of the electrical resistivity, in which the position of the minimum temperature changes from 95 K to 175 K, and a rise in the low temperature slope in the magnetic contribution (with -lnT dependence) from 21 μΩcm to 212 μΩcm is observed. In addition, the electronic coefficient of the specific heat increases almost twofold from 125 mJ/mol·K^2^ (x = 0.5) to 246 mJ/mol·K^2^ (x = 0.8). These changes are attributed to the variation of the distance between Yb and transition metals (Ni and Cu) along the series and the different electronic properties of the transition metals (Ni and Cu).

## 1. Introduction

The research on strongly correlated electron systems (SCES) occupies a relevant place within the areas of materials science research due to the rich phenomenology they present, connected directly to the understanding of the practical problem of mechanisms of high-temperature superconductivity [1]. Among them, Yb-based compounds have received special attention because of the variety of ground states which include magnetic, intermediate valence, and heavy fermion behaviors [2]. The nature of this ground state is established through a competition between the Kondo effect which favors a nonmagnetic ground state and the indirect magnetic intersite interaction between *4f* local moments via the conduction electrons (Ruderman–Kittel–Kasuya–Yosida (RKKY)) interaction [3]. The balance between these two interactions can be tuned through the *Jn_f_* parameter, where *J* is the coupling (exchange) between *4f* and conduction electrons, and *n_f_* is the density of states) with the application of pressure or by chemical substitution [1].

As a fine example, divalent Yb (nonmagnetic metal) under a pressure of 86 GPa becomes a superconductor before a stable trivalent (magnetic) state is reached, suggesting that magnetic instabilities may play a role in the appearance of superconductivity [4]. Another example is the antiferromagnetic YbCuGe, where the application of pressure induces a magnetic frustration in the triangular lattice of Yb ions of this compound [5]. On the other hand, the study of chemical substitution effects on the α-YbAlB_4_ system by Mn substitution at the Al site influences the intermediate valence state with the appearance of an antiferromagnetic transition at 20 K, a relatively high value for Yb compounds [6].

The present study focuses on the chemical substitution effects when Cu replaces Ni in the YbNiAl compound and the change in physical properties in the evolution toward YbCuAl. Both compounds belong to a large class of intermetallic compounds of the type RTX (R—rare earth, T—transition metal, X—p element) [7]; in particular, those belonging to the YbTX series have attracted considerable interest in the past [8]. The YbCuAl alloy has been the subject of exhaustive studies because of the mixed-valence characteristics of Yb [9,10,11,12,13]. Such a system with an intermediate valence (IV) state presents a characteristic temperature T_f_ above which a local description of the *4f* electrons seems applicable. However, below this temperature, an itinerant nonlocalized description is more adequate. According to the temperature dependence of the susceptibility for this alloy, the transition from the local (Curie–Weiss) high-temperature behavior to the nonlocal (T-independent Pauli-like) behavior occurs near T_f_ = 30 K [9,13]. The YbCuAl compound is also classified as a nonmagnetic CKS (concentrated Kondo system) with a high Kondo temperature value (T_K_ = 66 K), and an anomalous variation in temperature properties related to the formation of a narrow high-amplitude Abrikosov–Suhl resonance in the vicinity of the Fermi level [14]. Some studies have shown that, under a pressure of 8 GPa, the electrical resistivity curve shows an anomaly at 1 K, which has been interpreted as a sign of magnetic order [12]. Thus, the application of pressure, i.e., the unit cell volume reduction, favors the valence state 3^+^ of the *4f^13^* configuration of Yb ions and, hence, the magnetic ordered state. Alternatively, chemical substitution effects in the Yb_1−x_ Y_x_CuAl series of alloys have indicated the expansion of the unit cell volume when Y replaces Yb. Thus, that chemical strategy is suitable for the study of negative pressure effects on YbCuAl, resulting in an increase in the Kondo interaction with the increase in Y concentration [13]. On the other hand, another archetypal system is YbNiAl, where a strong competition between Kondo and RKKY interactions leads to the formation of a magnetic ordered HF ground state, with an antiferromagnetic transition at T_N_ = 3 K [15]. Clearly, it appears attractive to follow the evolution of the physical properties from the antiferromagnetic YbNiAl system to the intermediate valence (nonmagnetic ground state) of the pure YbCuAl, in order to unveil the effect of this large difference on the behavior of these two archetypal alloys, as seen in the YbInNi_4−x_Cu_x_ series of alloys [16]. Special attention is paid to how the changes in the properties are connected with the above-introduced *Jn_f_* parameter.

## 2. Materials and Methods

Samples of the YbNi_1−x_Cu_x_Al (with x = 0, 0.2, 0.5, and 1.0) series of alloys were prepared using a home-made arc-furnace, as used in the study of other Yb systems [13,16,17,18] and high-purity materials: Yb (99.9%), Ni (5 N), Cu (5 N), and Al (5 N) (Alfa-Johnson Matthey). Because of the high vapor pressure of Yb, small pieces of stoichiometric amounts of the materials were first pressed in a press pellet die with an excess of Yb (15–20%) to compensate for losses during fusion. The resulting ingots (of around 600 mg) were then melted three times to ensure homogeneity of the sample. During each step of melting, a loss of around 4–5% of Yb was observed. A subsequent annealing for 7 days of the resulting melting ingots at 750 °C was also carried out. In addition, reference (nonmagnetic) compounds YNi_1−x_Cu_x_Al (x = 0, 0.2, 0.5, and 1.0) were also prepared in the same setup in order to provide support to the analysis of the electrical resistivity data. X-ray diffraction patterns of the samples were recorded at 300 K in a Philips PW1710 diffractometer using CuK_α_ radiation (λ = 1.5418 Å) and a graphite secondary monocromator. The electrical resistivity (ρ) was measured using a standard four-probe dc method in a temperature range between 2 K and 300 K. The specific heat (c_p_) was measured in a small sample calorimeter between 5 K and 30 K, using the thermal relaxation method, with the same experimental setup used in the study of other Yb series of alloys [13,16,17].

## 3. Results

### 3.1. X-ray Diffraction

**Figure 1:**
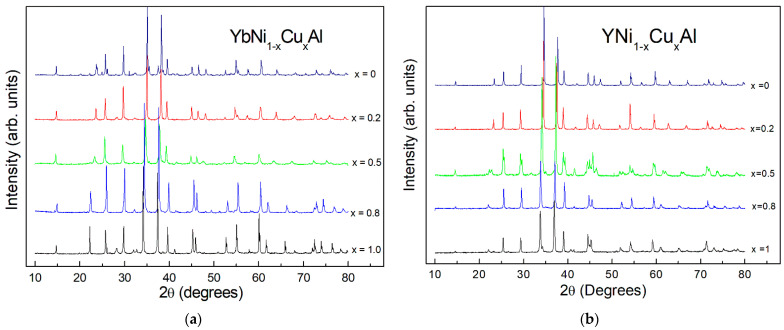
X-ray diffraction patterns of the YbNi_1_-_x_Cu_x_Al (**a**) and YNi_1_-_x_Cu_x_Al (**b**) series of alloys. All samples crystallize in a hexagonal ZrNiAl-type structure. The patterns of the different samples are shifted for comparison purposes.

The X-ray diffraction (XRD) patterns show the formation of the hexagonal ZrNiAl structure type for all samples of the series of YbNi_1−x_Cu_x_Al and YNi_1−x_Cu_x_Al alloys, as shown in Figure 1a,b, respectively. For comparison and clarity purposes, the diffractograms of the different samples are shifted.

The XRD patterns can be indexed according to the abovementioned type of structure, corresponding to the space group P-62m. The values of the crystallographic unit cell parameters were obtained using the Rietveld method implemented in the Fullprof suite programs [19]. The results of the Rietveld refinement of the XRD data are presented in Table 1 for the YbNi_1−x_Cu_x_Al series and in Table 2 for the YNi_1−x_Cu_x_Al series. The refinements show a reasonable reliability with standard Bragg factors (R_B_) between 10% and 15%. The lattice parameters calculated for YbCuAl (x = 1) were a = 6.9360 (6) Å and c = 4.0044 (4) Å, while those calculated for YbNiAl (x = 0) were a = 6.9610 (6) Å and c = 3.7743 (4) Å. These are in excellent agreement with those reported in the literature [9,10,15]. The unit cell volume values were also calculated (V = a^2^ c sen60°, for hexagonal lattices), as presented for both series (Table 1 and Table 2). In the case of the Yb alloys, a small fraction (less than 5%) of YbAl_2_ [20] and antiferromagnetic Yb_2_O_3_ (T_N_ = 2.1 K) was found. The nonmagnetic characteristics of YbAl_2_ and the very low Néel transition (T_N_ = 2.1 K) of the antiferromagnetic Yb_2_O_3_ did not affect the interpretation of the properties of the main phase. It should be noted that the presence of a small fraction of Yb_2_O_3_ is commonly found in Yb alloys, appearing even in single crystals [13,21,22].

**Figure 2:**
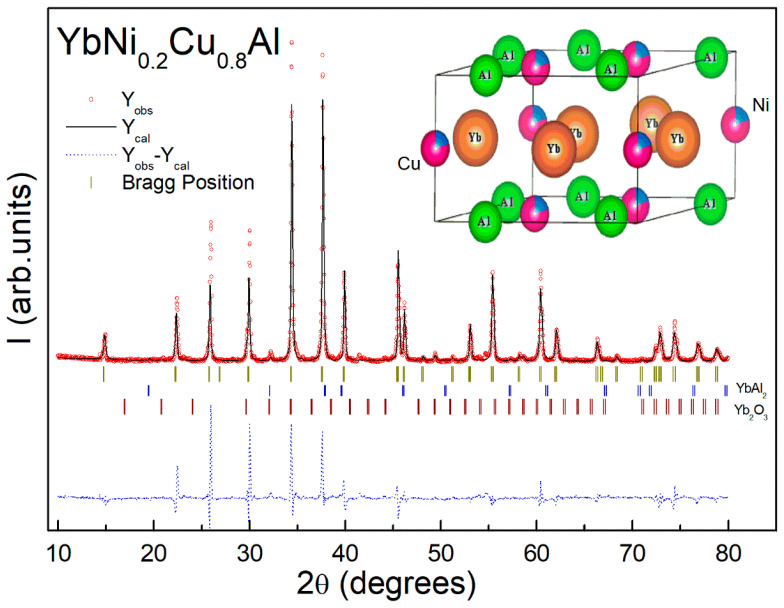
XRD pattern and Rietveld refinement in a representative Yb sample with x = 0.8. A good reliability of the fit is observed, with a standard Bragg factor (R_B_ = 10.4%). Bragg positions (vertical markers) of the main phase and impurity phases (YbAl_2_ and Yb_2_O_3_) are also provided. The scheme of the typical structure (inset) with the distribution of the different atoms is shown.

As an example, the Rietveld refinement of x = 0.8 is presented in Figure 2. A good reliability of the fit is obtained with a Bragg factor (R_B_ = 10.4%). Vertical markers indicate the positions of the peaks of the main phase (96.5 (7)%) and impurity phases: YbAl_2_ (1.2%) and Yb_2_O_3_ (2.3%). The crystallographic structure of x = 0.8 consisted of planes of Yb, Ni, and Cu atoms between two others of Al, Ni, and Cu, with the Cu and Ni statistically distributed according to the ratio Ni (20%) and Cu (80%) (see inset of Figure 2).

**Figure 3:**
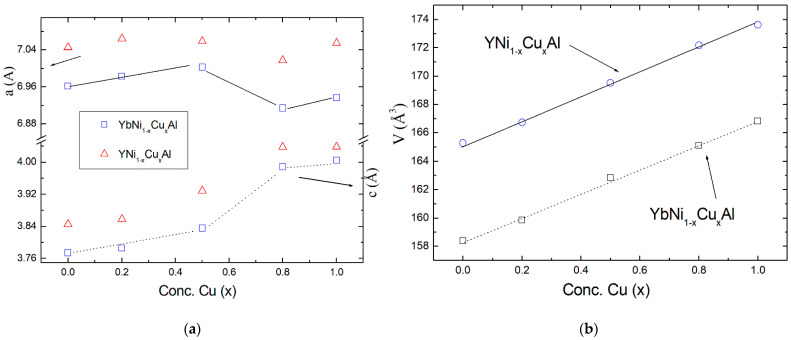
(**a**) Lattice a, c parameters vs. Cu concentration (x) in the series of YbNi_1−x_Cu_x_Al and reference YNi_1−x_Cu_x_Al compounds. There is an abrupt deviation from the linear behavior between x = 0.5 and 0.8. (**b**) Unit cell volume vs. Cu concentration (x) variation following a linear behavior. Error bars (not indicated) are within the size of data points (see Table 1 and Table 2).

For the sake of clarity, the variation of the lattice parameters and the unit cell volume along the series as a function of the Cu concentration (x) is presented in Figure 3b. Interestingly, despite a linear variation (increase) of the unit cell volume with increasing Cu concentration (x), as found in other chemically substituted series of alloys [9,13,16,17], here, an anomalous variation of the lattice parameters was found (see Figure 3a). Indeed, for x = 0.8 and x = 1, a deviation from the general trend of x = 0, 0.2, and 0.5 alloys is observed. This anomaly cannot be attributed to changes in the valence with the Cu dilution upon going to the intermediate valence system YbCuAl, as the same behavior is found in the nonmagnetic counterpart YNi_1−x_Cu_x_Al series of alloys, as depicted in Figure 3. It is described below that the anomalous variation of the lattice parameters may have implications on the physical properties.

### 3.2. Electrical Resistivity

**Figure 4:**
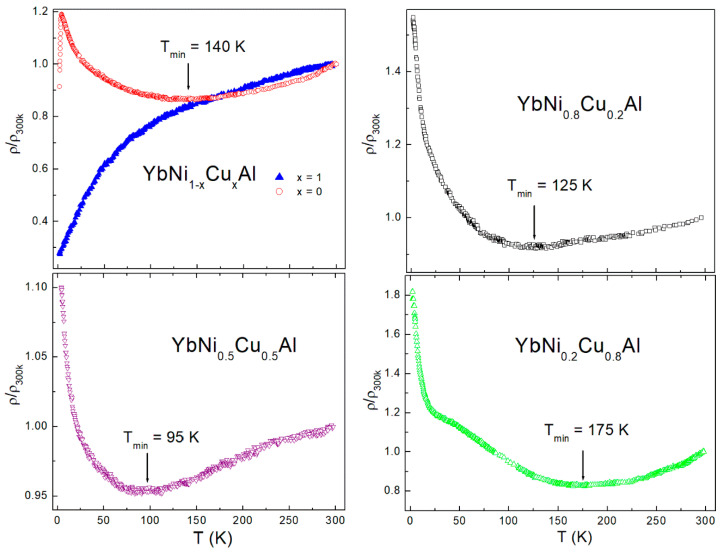
Temperature dependence of the electrical resistivity of the YbNi1−xCuxAl series of alloys in normalized form (ρ/ρ_300Κ_). The minimum in the electrical resistivity associated with the Kondo effect is marked with an arrow, except for x = 1, where a Kondo lattice behavior is observed.

In Figure 4, the results of the measurements of the electrical resistivity for all samples normalized to the value at 300 K (ρ/ρ_300K_) are presented. The sample with x = 1 displays a different behavior with respect to the rest of the samples, with a characteristic decrease in the resistivity at lower temperatures according to a Kondo lattice system [13]. The remaining samples show a typical single-impurity Kondo behavior with the presence of a minimum and an abrupt increase in the resistivity on going to lower temperatures. The minimum associated with the Kondo effect is indicated by an arrow (and its temperature value, T_min_). Interestingly, there is a trend marking a decrease from x = 0 to x = 0.5, which is broken for x = 0.8. The possible causes of this behavior are discussed below.

**Figure 5:**
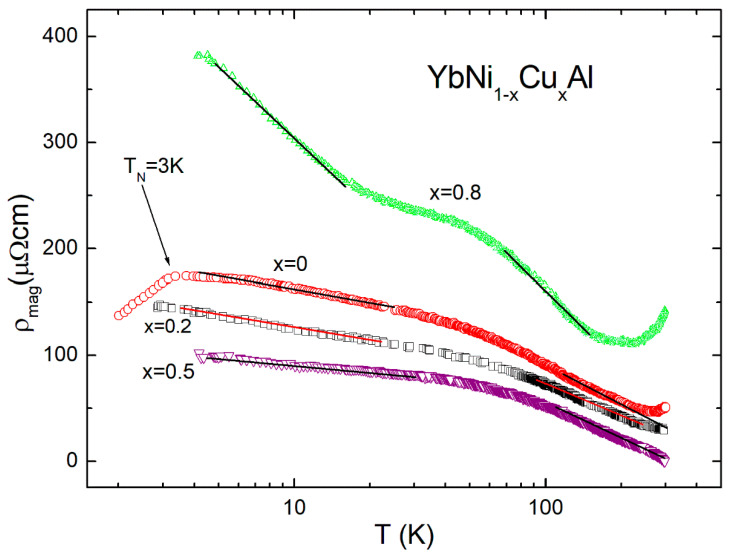
Magnetic contribution vs. temperature (in logarithmic scale) obtained by subtraction of the experimental data in YbNi_1−x_Cu_x_Al alloys using reference YNi_1−x_Cu_x_Al series. Two regions with −lnT dependence are observed.

The electrical resistivity of isostructural compounds YNi_1−x_Cu_x_Al shows a typical metallic behavior according to the Bloch–Grunëisen law, similar to that reported for YCuAl (x = 1) alloy [13]. To extract the magnetic contribution ρ_mag_ vs. temperature in the Yb-based series YbNi_1−x_Cu_x_Al, the resistivity of the nonmagnetic Y-based counterparts was subtracted, as presented in Figure 5. The resulting curves display two regions with −lnT dependence, characteristic of a Kondo interaction in the presence of crystalline field effects [16,17,23,24]. In hexagonal symmetry, the eightfold degenerate state of the Yb^3+^ ion with J = 7/2 is expected to split into four doublets [25]. The ratio of the high- and low-temperature slopes for x = 0.5 compound is around 5.1. This fact is consistent with a doublet ground state and a doublet excited state according to the Cornut–Coqblin model developed for Ce compounds [16,17,23,24]. However, for x = 0.8, the ratio is around 1.2, which indicates that this model could partially fail for the case of Yb-based alloys or additional effects must be taken into account [17,23]. The presence of crystalline field effects is clearly visible with a hump around 60 K (regions where the two slopes separate each other), which further indicates the splitting of the first excited state around 90 K [24]. For x = 0, a maximum around 3 K with a decrease below this temperature is associated with the AFM order [15].

### 3.3. Specific Heat

**Figure 6:**
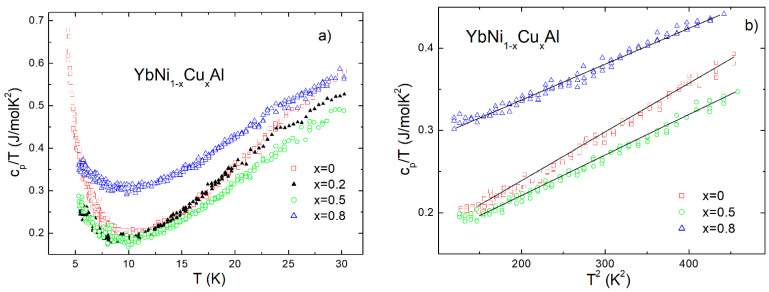
(**a**) c_p_/T vs. temperature dependence in the YbNi_1−x_Cu_x_Al series of alloys. The onset of competition between magnetic correlations and the Kondo effect below the minimum around 10 K is observed. (**b**) Plot of c_p_/T vs. T^2^ dependence showing a linear behavior in the temperature range between 11 K and 20 K.

In Figure 6a, a plot of c_p_/T vs. T is shown for the samples of the series YbNi_1−x_Cu_x_Al in the low temperature range (T < 30 K).

The minimum around 10 K indicates the interplay of the competition between RKKY interaction and the Kondo effect on going to low temperatures [17,23], as indicated by electrical resistivity measurements (Figure 4 and Figure 5). Above this temperature and up to 20 K, the c_p_/T vs. T^2^ dependence displays a linear behavior, which is in agreement with a c_p_ ≈ **γ**T + βT^3^ specific heat dependence for metals [13] (**γ** and β are related to the electronic and phonon contributions, respectively) as shown in Figure 6b. For the sake of clarity, the data of the x = 0.2 sample are not shown. Neglecting the influence of crystalline field effects, the estimate of the electronic coefficient gives close values around 117 mJ/mol·K^2^ for x = 0, 0.2, and 0.5. However, an abrupt **γ**-increase for x = 0.8 (246 mJ/mol·K^2^) is observed. The probable causes of this striking behavior are discussed in detail in the next section.

## 4. Discussion

The joint analysis of the X-ray diffraction, electrical resistivity, and specific heat results on the YbNi1−xCuxAl alloys shown above allows discussing in detail the overall behavior of the samples. It is possible to relate the changes in the electrical transport and the thermal properties with the behavior of the lattice parameters upon Cu substitution (summarized in Table 3). As presented in Figure 3, there is an abrupt change between x = 0.5 and 0.8 in the lattice parameters, which can be basically associated with the Cu substitution by Ni and not to the change in the valence, because a similar change also appears in the nonmagnetic counterpart YNi_1−x_Cu_x_Al series. These variations in the crystallographic structure are reflected in the other properties. For instance, the position in temperature of the minimum in the electrical resistivity, a fingerprint of the Kondo effect, (T_min_, see Figure 4 and Table 3) decreases with the increase in Cu content from x = 0 to x = 0.5. Such a tendency is broken for x = 0.8, where an abrupt increase from 95 K to 175 K is observed. A similar trend can be deduced from the low-temperature slope (parameter *C* in Table 3), associated with Kondo behavior with a characteristic −lnT dependence. This is deduced from the analysis of the magnetic contribution of the electrical resistivity on the YbNi_1−x_Cu_x_Al series of alloys (see Figure 5).

Moreover, the estimate of the electronic coefficient from the analysis of the specific heat data, particularly from the c_p_/T vs. temperature curves (Figure 6b), is also in agreement with the tendency observed in the two mentioned parameters: T_min_ and *C* (Table 3). Additionally, this increase in **γ** value may also be due to the influence of the electronic effects, as the substitution of Ni by Cu leads to an increase in the number of conduction electrons [16,26,27].

**Figure 7:**
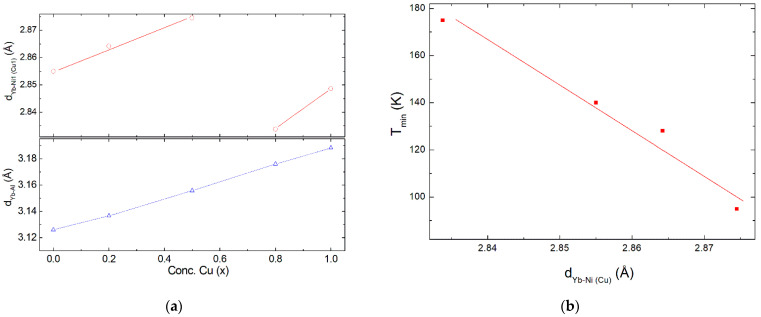
(**a**) Dependence of the distance between Yb and transition-metal atoms (Ni and Cu) (d_Yb–Ni(Cu)_) and Al atom (d_Yb–Al_) as a function of the Cu concentration (x). (**b**) Temperature of the minimum in the electrical resistivity vs. d_Yb–Ni(Cu)._ A simple correlation between both parameters is found.

Additionally, the change in the hybridization between *4f* electrons with the *d*-conduction band of transition metals Ni and Cu or *p*-band of Al must also play a significant role, as observed in CeNi_1−x_Cu_x_ series of alloys [26,27]. According to the Wolf–Schrieffer transformation, a direct relation can be established between the Kondo coupling constant (*J*) of *4f* and conduction electrons and the hybridization [14]. Moreover, as the Kondo temperature is related to the coupling constant (*J*) and the density of states at the Fermi level (*n*_f_) as T_K_ ≈ exp(−1/Jn_f_), an increase in the hybridization or in the *Jn_f_* product leads to an increase in the Kondo interactions. Since the strength of this hybridization between *4f* and conduction electrons in YbNi_1−x_Cu_x_Al series of alloys depends on the distance between Yb and transition metal (Ni, Cu) or Al atoms, it is productive to follow how these distances change with the variation of the Cu concentration (x) along the series. These distances can be derived from the variation of the lattice parameters, obtained by the analysis of the X-ray diffraction data (Table 1 and Table 2, Figure 3). In this sense, in Figure 7a, the dependence of the distance between Yb and transition metals (*d*_Yb–(Ni,Cu)_) and Al (*d*_Yb–Al_) as a function of the Cu concentration (x) is presented. It is observed that *d*_Yb–Al_ varies linearly with the change in the Cu concentration for all the studied samples between x = 0 and x = 1, just as the unit cell volume does (Figure 3). However, *d*_Yb–(Ni,Cu)_ suffers an abrupt change around x = 0.7, which is similar to that observed in the parameters presented in Table 3. This indicates that a direct relation between the change un this distance *d*_Yb–(Ni,Cu)_ and some parameters reflecting the variation in the Kondo interactions with the Cu alloying can be established, i.e., the minimum of the electrical resistivity (T_min_), as depicted in Figure 7b. This further indicates that, despite the substitution of Ni by Cu potentially provoking an increase in the density of states as a result of electronic effects [16,26,27], there is also a contribution of the structural changes along the series basically affecting the *4f–d* hybridization. Therefore, the strength of Kondo interactions is equally affected. In fact, this is supported by the change in the low-temperature slope (*C*) in the magnetic contribution to the electrical resistivity (Table 3) since *C* ≈ *J*^3^n_f_^2^ [17,23,24]. This indicates that the evolution from a magnetic behavior for x = 0 (Ni-side) to nonmagnetic HF state for x = 1 (Cu-side) is favored with the increase in Cu concentration (x). This situation is similar to that observed in other Ni–Cu-substituted series of alloys, such as Yb(Cu_1−x_Ni_x_)_2_Si_2_, where the magnetism is favored for high Ni concentrations, and the Kondo temperature is found to decrease on going to the Ni side [28].

Another example was found when Cu replaces Ni in ferromagnetic YbInNi4 (YbInNi_4−x_Cu_x_ series). In this case, the magnetism disappears on going to the Cu side [16]. Thus, considering the above results, for Yb systems, it can be concluded that the RKKY interaction is favored in Ni compounds displaying a magnetic ordering, whereas, in Cu compounds, a nonmagnetic state would be dominant [18]. On the other hand, in Ce compounds, an inverse situation is found; a magnetic state is favored for Cu-rich compounds and the nonmagnetic state is favored for the CeNi one, as observed, for instance, in the studies of CeNi_1−x_Cu_x_ [26,27] and CeNi_1−x_Cu_x_Al [29] series of alloys. In particular, in CeNi_1−x_Cu_x_ series, a transition from the nonmagnetic state in CeNi to ferromagnetic state for 0.3 ≤ x < 0.8 and antiferromagnetic state for CeCu (x = 1) was reported [26]. In the CeNi_1−x_Cu_x_Al series of alloys with x = 0, 0.05, 0.1, 0.2, and 0.3, a smooth transition between the mixed-valence (nonmagnetic) state of CeNiAl to a trivalent state (Ce^3+^) with the Ni substitution by Cu was observed [29]. It is worth commenting that, in contrast to these chemically substituted studies in Ce alloys and in YbInNi_4−x_Cu_x_ series [16], where both the lattice parameters and unit cell volume evolve continuously, the present YbNi_1−x_Cu_x_Al series showcases an abrupt variation of the lattice parameters with the Cu substitution.

## 5. Conclusions

A study on the influence of chemical substitution effects when Cu replaces Ni in the antiferromagnetic YbNiAl alloy (YbNi_1−x_Cu_x_Al series of alloys) was performed. The analysis of the X-ray diffraction data indicates an anomalous change in the unit cell parameters around x = 0.7, despite a common linear overall behavior of the unit cell volume. The presence of such abrupt changes contrasts the observations in most systems where a chemical substitution is performed [9,13,16,17,18]. In particular, the variation of the distance between Yb and transition metals (Ni,Cu) (*d*_Yb–(Ni,Cu)_) is indicated as the main cause affecting the variation of the minimum of the electrical resistivity (T_min_), of the low-temperature slope in the magnetic contribution to the electrical resistivity (*C*), and of the electronic coefficient (γ) of the specific heat as a function of the Cu concentration (x). This clearly indicates that, although an influence of electronic effects with the increase in the density of states when Cu replaces Ni is surely present, the hybridization between *4f* and *d*-band conduction electrons would play the main role in changes of the physical properties along the YbNi_1−x_Cu_x_Al series. Thus, the evolution from the magnetic behavior of YbNiAl to a nonmagnetic HF state of the YbCuAl system would be driven by a strong competition between the RKKY and Kondo effect, depending basically on the *4f–3d* hybridization, due to the variations in the crystallographic structure. In this context, it would be interesting to conduct electron spin resonance measurements to analyze the variation of the Yb-conduction electron exchange parameter J_Yb_, similar to what has been done in the Y_1−x_Yb_x_InNi_4_ series of alloys [18]. Furthermore, it would be interesting extend this kind of study to other Yb systems, for instance, in the YbNi_2_ binary alloy displaying ferromagnetism and HF behavior [30], through the YbNi_2−x_Cu_x_ series of alloys.

## Figures and Tables

**Table 1:** Lattice parameters (a, c) and volume (V) as calculated by Rietveld refinements of the X-ray diffraction data of the YbNi_1−x_Cu_x_Al series of alloys.

YbNi_1−x_Cu_x_Al	a (Å)	c (Å)	V (Å^3^)
x = 0	6.9610 (6)	3.7743 (4)	158.38 (4)
x = 0.2	6.9823 (4)	3.7863 (3)	159.86 (3)
x = 0.5	7.002 (1)	3.8355 (8)	162.85 (8)
x = 0.8	6.9139 (5)	3.9883 (3)	165.11 (4)
x = 1.0	6.9360 (6)	4.0044 (4)	166.83 (5)

**Table 2:** Lattice parameters (a, c) and volume (V) as calculated by Rietveld refinements of the X-ray diffraction data of the YNi_1−x_Cu_x_Al series of alloys.

YNi_1−x_Cu_x_Al	a (Å)	c (Å)	V (Å^3^)
x = 0	7.0451 (5)	3.8450 (3)	165.28 (4)
x = 0.2	7.0641 (5)	3.8582 (3)	166.73 (4)
x = 0.5	7.0588 (8)	3.9287 (6)	169.53 (6)
x = 0.8	7.0169 (6)	4.0377 (5)	172.17 (5)
x = 1.0	7.0461 (5)	4.0382 (3)	173.63 (4)

**Table 3:** Dependence on the Cu concentration (x) of some parameters extracted from the electrical resistivity and specific heat results in the series of YbNi_1−x_Cu_x_Al alloys.

YbNi_1−x_Cu_x_Al	T_min_ (K) (±10%)	*C* (μΩcm) (±1%)	γ (mJ/mol·K^2^) (±1%)
x = 0	140	46	113
x = 0.2	128	39	114
x = 0.5	95	21	125
x = 0.8	175	212	246
x = 1	-	-	340

## Data Availability

Not applicable.
